# Sarcoplasmic Reticulum Calcium Release Is Required for Arrhythmogenesis in the Mouse

**DOI:** 10.3389/fphys.2021.744730

**Published:** 2021-10-12

**Authors:** Andrew G. Edwards, Halvor Mørk, Mathis K. Stokke, David B. Lipsett, Ivar Sjaastad, Sylvain Richard, Ole M. Sejersted, William E. Louch

**Affiliations:** ^1^Institute for Experimental Medical Research, Oslo University Hospital, University of Oslo, Oslo, Norway; ^2^Department of Pharmacology, University of California, Davis, Davis, CA, United States; ^3^K.G. Jebsen Centre for Cardiac Research, University of Oslo, Oslo, Norway; ^4^Department of Cardiology, Oslo University Hospital, Oslo, Norway; ^5^Université de Montpellier, INSERM, CNRS, PhyMedExp, Montpellier, France

**Keywords:** early afterdepolarizations (EADs), delayed afterdepolarizations, species, triggered activity, repolarization

## Abstract

Dysfunctional sarcoplasmic reticulum Ca^2+^ handling is commonly observed in heart failure, and thought to contribute to arrhythmogenesis through several mechanisms. Some time ago we developed a cardiomyocyte-specific inducible SERCA2 knockout mouse, which is remarkable in the degree to which major adaptations to sarcolemmal Ca^2+^ entry and efflux overcome the deficit in SR reuptake to permit relatively normal contractile function. Conventionally, those adaptations would also be expected to dramatically increase arrhythmia susceptibility. However, that susceptibility has never been tested, and it is possible that the very rapid repolarization of the murine action potential (AP) allows for large changes in sarcolemmal Ca^2+^ transport without substantially disrupting electrophysiologic stability. We investigated this hypothesis through telemetric ECG recording in the SERCA2-KO mouse, and patch-clamp electrophysiology, Ca^2+^ imaging, and mathematical modeling of isolated SERCA2-KO myocytes. While the SERCA2-KO animals exhibit major (and unique) electrophysiologic adaptations at both the organ and cell levels, they remain resistant to arrhythmia. A marked increase in peak L-type calcium (*I*_CaL_) current and slowed *I*_CaL_ decay elicited pronounced prolongation of initial repolarization, but faster late repolarization normalizes overall AP duration. Early afterdepolarizations were seldom observed in KO animals, and those that were observed exhibited a mechanism intermediate between murine and large mammal dynamical properties. As expected, spontaneous SR Ca^2+^ sparks and waves were virtually absent. Together these findings suggest that intact SR Ca^2+^ handling is an absolute requirement for triggered arrhythmia in the mouse, and that in its absence, dramatic changes to the major inward currents can be resisted by the substantial K^+^ current reserve, even at end-stage disease.

## Introduction

Dysfunctional sarcoplasmic reticulum (SR) Ca^2+^ handling is known to destabilize cardiac electrophysiology in a broad range of arrhythmogenic diseases, from rare channelopathies (Priori et al., 2001, 2002) to prevalent acquired diseases such as heart failure (Pogwizd et al., 2001; Pogwizd and Bers, 2002). However, the role of the SR as an intracellular Ca^2+^ store able to drive cardiac contraction is fundamental to normal function of the heart, and evolutionary processes have carefully balanced the electrical stability of cardiac excitation-contraction (EC) coupling with the requirement for a high-gain intracellular Ca^2+^ release system. This balance has important implications for arrhythmia mechanisms in the heart, and these can be clearly illustrated by considering differences across mammalian species.

In healthy large mammals, approximately 30% of the Ca^2+^ that fuels cardiac contraction is obtained from L-type Ca^2+^ current (*I*_CaL_)-mediated Ca^2+^ influx (Shannon et al., 2004; Fearnley et al., 2011), and this can increase to near 50% in chronic diseases such as heart failure (Pogwizd and Bers, 2002). Such large transmembrane Ca^2+^ fluxes are permitted by a prolonged action potential (AP) plateau, which itself results from a relatively delicate balance of inward and outward currents in large mammals (Weiss et al., 2010). These characteristics of EC coupling in large mammals tend to favor repolarization instabilities, and potentiation of *I*_CaL_ by frequency, neurohormonal stimuli, and genetic abnormalities are well known to destabilize repolarization in humans (Piot et al., 1996; Splawski et al., 2004, 2005; Sato et al., 2009; Tran et al., 2009). Indeed, it is for exactly this reason that significant investments have been made to establish and study large animal models of human arrhythmogenic diseases thought to result from repolarization abnormalities (Brunner et al., 2008; Koren, 2009).

In contrast, the structure of murine EC coupling pushes the mouse heart toward arrhythmogenic mechanisms that rely upon unstable SR Ca^2+^ handling, and away from mechanisms that result directly from repolarization instabilities due to *I*_Ca,L_ or other surface membrane currents. In particular, the large and rapidly activating outward K^+^ currents in the mouse and rat permit only a very brief AP. This both limits the degree to which *I*_CaL_ can contribute to contractile Ca^2+^ and necessitates a larger contribution from the SR Ca^2+^ store (∼92%) (Bers, 2001). This promotes instability in both systolic and diastolic Ca^2+^ handling. Indeed, the mouse has proven to be a very useful model for studying arrhythmia phenotypes resulting from aberrant spontaneous SR Ca^2+^ release (Lehnart et al., 2008; Kashimura et al., 2010; Bai et al., 2013), and we have also shown that unstable triggered Ca^2+^ release recruits unique EAD dynamics in mice (Edwards et al., 2014). *In vivo* studies utilizing simultaneous ECG and surface mapping (monophasic AP or optical mapping) have suggested that the dominant mechanisms of arrhythmia in these mice are focal activity (perhaps originating from DADs in the His-Purkinje network), and APD alternans driven by aberrant SR Ca^2+^ handling (Lehnart et al., 2006; Cerrone et al., 2007). Together, these characteristics have led us to hypothesize that destabilized Ca^2+^ handling is a fundamental requirement for triggered arrhythmia in the mouse, and that in the absence of intact SR Ca^2+^ release, even a dramatically altered balance of sarcolemmal currents is not sufficient to elicit arrhythmia in response to commonly applied neurohormonal challenge.

To interrogate this hypothesis, we have examined *in vivo* and cellular arrhythmogenesis in a conditional SR Ca^2+^ ATPase type 2 (SERCA2) knockout mouse (KO). These mice progress to contractile failure in 7–10 weeks and display remarkable adaptations to the rapid loss of cardiac SR Ca^2+^ reuptake (Andersson et al., 2009; Liu et al., 2011; Li et al., 2012; Swift et al., 2012; Land et al., 2013). Most prominently, the Ca^2+^ fluxes responsible for supporting myofilament activation shift from the SR to the sarcolemma, with several fold increases in the inward currents attributable to *I*_CaL_ and forward mode Na^+^-Ca^2+^ exchange (*I*_NaCa_). In combination, these adaptations constitute a genetic model of extreme loss of *I*_Ca__L_ control in the mouse, and present a unique opportunity to directly dissect the role of sarcolemmal versus intracellular mechanisms in murine arrhythmia.

At end-stage life we found SERCA2 KO mice are remarkably resistant to arrhythmia, both at the cellular level and in intact conscious animals. This resistance exists in the face of large increases in both *I*_Ca__L_ and Na^+^-Ca^2+^ exchange, both of which contribute to marked prolongation of the AP and are ordinarily interpreted as strongly proarrhythmic changes. In combination, these data strongly suggest that SR Ca^2+^ release is required for cellular arrhythmogenesis and tissue-level triggered events in the mouse.

## Materials and Methods

All experiments were performed in accordance with the Norwegian Animal Welfare Act, which conforms to NIH guidelines (NIH publication No 85-23, revised 1996).

### Mouse Model

The SERCA2 knock out mouse (KO) has previously been described and studied in detail as a model of contractile failure (Andersson et al., 2009; Liu et al., 2011; Li et al., 2012; Swift et al., 2012; Land et al., 2013). Briefly, cardiac-specific SERCA2 KO excision was achieved by tamoxifen activation of Cre-recombinase (α-MHC driven MerCreMer) via a single tamoxifen injection at 8–12 weeks of age. Here we studied these animals near to their mean age of death at 7 weeks after tamoxifen injection, when they exhibit pronounced contractile dysfunction, and markedly reduced cardiac output (Andersson et al., 2009). Age-matched flox/flox mice (FF) were used as controls.

### Electrocardiography

Telemetry transmitters (Physiotel ETA-F10, Data Sciences International, St. Paul, MN, United States) were used to record electrocardiograms in freely moving animals. Implantation was performed 6 weeks after tamoxifen injection, and as previously described (Manotheepan et al., 2016). Intraperitoneal Xylazine hydrochloride and ketamine hydrochloride were administered in combination with isoflurane inhalation (1–4%) during the procedure, and subcutaneous buprenorphine was given toward the end of the procedure for postoperative analgesia. The transmitters were placed subcutaneously in the dorsal thoracolumbar region, and stabilized by ligatures to the dorsal muscles. Leads were attached to the pectoral muscles by ligatures in the upper left and lower right pectoral regions. Electrocardiograms were recorded after 7 days recovery from surgery, and recordings were analyzed for heart rate as well as ECG parameters during baseline conditions, and after a subsequent intraperitoneal injection of adrenalin (Epi, 0.5mg/kg). Analysis was performed manually and in fully-blinded fashion by the same experienced technician using both proprietary software (Matlab version 2013b, The Mathworks, Natick, MA, United States), and Ponemah (Data Sciences International, St. Paul, MN, United States). Steady state ECG analyses were performed by applying conventional definitions for all intervals. Of note, because the mouse often exhibits a pronounced J-wave we have defined QRS width (duration) as the time from first negative deflection after the P-wave to the first time after the S-wave minimum at which the ascending voltage signal crosses the isoelectric line. Rate dependent QT correction was performed by the Bazzet formula:


$$Q\,T\,c=\frac{Q\,T}{\sqrt{R\,R}}$$

Premature ventricular complexes (PVCs) were manually identified, and episodes of ventricular tachycardia (VT) were defined as 4 or more PVCs occurring in sequence. Runs of VT were defined as non-sustained (NSVT) if they lasted less than 20-s, and as sustained (SVT) if longer.

### Cell Isolation

Cardiomyocytes were isolated from ventricles of FF and KO mice similarly to our prior studies (Ottesen et al., 2015; Manotheepan et al., 2016). Briefly, mice were isoflurane-anesthetized (98% oxygen, 2.0% isoflurane), and euthanized by cervical dislocation. Excised hearts were first rinsed in ice-cold isolation buffer containing (mM): 130 NaCl, 25 Hepes, 22 D-glucose, 5.4 KCl, 0.5 MgCl_2_, 0.4 NaH_2_PO4 (pH 7.4). Each heart was then retrograde-perfused under constant flow conditions (3 ml/min) with warmed (37°C) isolation buffer for 4 min, and then with the same buffer supplemented with 400 U/ml collagenase Type II (Worthington Biochemical Corporation, Lakewood, NJ, United States) and 0.015 mmol/L Ca^2+^. After 10 min of enzyme perfusion, hearts were cut down and the left ventricle was removed, diced, and triturated in collagenase-free isolation buffer including 1% BSA and 0.02 U/L deoxyribonuclease I (Worthington), again at 37°C. The resulting cell suspension was filtered (200 μm nylon mesh) and sedimented, the cell pellet was washed in isolation buffer supplemented with 1% BSA, and Ca^2+^ was progressively reintroduced (0.05, 0.1, 0.2, and 0.5 mmol/L). The Ca^2+^-tolerant cardiomyocytes were stored at room temperature until use, which occurred within 8 h of isolation.

### Cell Electrophysiology

Single rod-shaped cardiomyocytes with clear striations were patch-clamped in whole-cell configuration using Axoclamp 2A and 2B amplifiers. Borosilicate patch-pipettes had resistances of 2–3 MOhms with the corresponding internal and external solutions. We used identical solutions for current clamp AP recordings and end-pulse K^+^ currents in voltage clamp, where the internal solution contained (in mM: 120 K-Asp, 25 KCl, 0.5 MgCl_2_, 6 NaCl, 4 K_2_-ATP, 0.06 EGTA, 10 HEPES, and 10 D-Glucose, pH corrected to 7.2 with KOH), and external (in mM): NaCl 140, HEPES 5, KCl 5.4, MgCl_2_⋅6H_2_O 0.5, Glucose 5.5, NaH_2_PO_4_H_2_O 0.4, CaCl_2_ 1. For voltage clamp recordings of the transient outward potassium current (*I*_to_), the internal solution contained (in mM) 110 K-Asp, 20 KCl, 0.5 MgCl_2_, 4 K_2_-ATP, 5 EGTA, 5 HEPES, and 10 D-Glucose - pH 7.2 with KOH, with a Na^+^ and Ca^2+^ free external solution (mM): 140 CholineCl, 1 CdCl_2_, 0.5 MgCl_2_, 5 HEPES, 5.5 Glucose, 5.4 KCl, pH 7.2 with KOH. For *I*_Ca,L_ the internal solution was (mM) 130 CsCl, 0.33 MgCl_2_, 4 Mg-ATP, 0.06 EGTA, 10 HEPES and 20 tetraethylammonium chloride - pH 7.2 with CsOH, and the bathing solution included (mM) 135 NaCl, 20 CsCl, 1 MgCl_2_, 10 glucose, 10 HEPES, 1 CaCl_2_, and 4 4-AP (pH 7.4).

### Electrophysiologic Protocols

Cells were superfused with external solution in all experiments, and all current measurements were made during step-pulse protocols at 1 Hz. Following ten 50-ms conditioning pulses (also 1 Hz) to 0 mV from a holding potential of −70 mV, *I*_CaL_ was elicited by a 200-ms depolarizing voltage step (Mørk et al., 2009). Test potentials ranged from −40 to +50 mV with 10-mV increments from a post-train holding potential of −40 mV (to inactivate *I*_Na_). Current amplitude was measured as the difference between peak inward current and steady-state current at the end of the test pulse. *I*_to_ (peak *I*_K_) was elicited by 300-ms steps to test potentials between −40 and +60 mV in 10-mV increments from a holding potential of −70 mV (Louch et al., 2010a). Peak *I*_K_ was calculated as the peak outward current less the steady-state end pulse current from these 300-ms steps. The pedestal or plateau component of *I*_K_ (*I*_K__p_) was calculated as the mean of the final 20-ms of 500-ms test-pulse steps between −100 and +60 mV, again at 10 mV increments from a –70 mV holding potential. All currents were normalized to capacitance, which was calculated as the integral of the transient current response to a 10-mV hyperpolarizing step (150 ms) from a holding potential of −70 mV.

Action potential waveforms were also recorded at 1 Hz in all experimental conditions, and at least 20 sequential beats were allowed to achieve steady state. In experiments assessing EAD and DAD susceptibility, Iso-containing superfusate (1 μM) was applied after 30-s of baseline recording, and maintained for at least 3 min further.

### Mathematical Modeling

The computational models used here are modifications of those published by Li et al. (2012). Briefly, both the FF and KO models were constructed on the basis of extensive data collected in the FF and KO mice at the same age and time after SERCA gene excision as studied here. Our modifications here were made sequentially to establish the requirements for the observed AP changes accompanying SERCA-KO. Thus, we describe them in that sequence in the results section. All parameter changes and details for code access are provided in the Supplementary Material.

### Statistics

Steady state electrocardiographic parameters were assessed by repeated measures ANOVA (RMANOVA: Genotype × Epi) with repeated measures for Epi. ECG arrhythmia outcomes (PVC and NSVT frequency) were non-normally distributed, and interrogated by Kruskal-Wallis rank sum test after being separated into baseline and Epi recordings. Similarly, skewed data for EAD frequency were assessed via the Kruskal-Wallis test, and the Fisher exact test was used to determine differences in EAD incidence. Peak *I*_K_, *I*_K__p_, *I*_CaL_, and AP durations at 20, 50, 70, and 90% repolarization were all tested by RMANOVA where step potential was repeated for current measurements, and treatment conditions (Ca^2+^ concentration, Nifedipine, or Isoproterenol) were repeated for AP recordings.

For all RMANOVAs, the Holm-Sidak test was used to identify pairwise effects *post hoc*. In the event that the sphericity assumption was not valid, RMANOVA was replaced by the Mann-Whitney-Wilcoxon test. Significant effects were defined at *p* < 0.05, and *p*-values are explicitly stated for all marginal results (0.05 ≤ *p* ≤ 0.1). All statistical analyses were performed either with SigmaPlot (Systat Software Inc., CA, United States), or R software (version 3.2.1, The R Foundation of Statistical Computing).

## Results

### SERCA2-KO Mice Exhibit Unique but Stable Changes in Global Cardiac Electrophysiology

**FIGURE 1:**
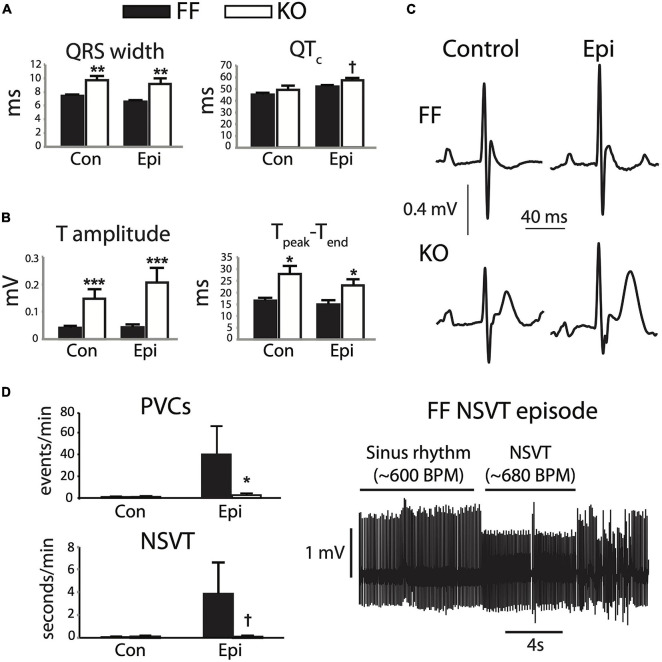
SERCA2-KO mice exhibit unique alterations in global cardiac electrophysiology. Telemetered ECG recordings from 8 animals in each group were first analyzed for steady state characteristics from periods exhibiting only normal sinus activation sequence **(A,B)**. KO mice showed increased QRS width (**A**, left), and marked increases in T-wave amplitude and duration **(B)**. **(C)** Typical sinus waveforms recorded from FF and KO animals. Ectopic activity was also analyzed **(D)**, and KO animals surprisingly exhibited a reduced frequency of total ectopy (PVC frequency, top left), and a trend for fewer runs of non-sustained VT (bottom left), during Epi challenge. All panels: ^†^*p* < 0.1 KO vs. FF, *p < 0.05 KO vs. FF, **p < 0.01 KO vs. FF, ***p < 0.001 KO vs. FF. Data are means ± SEM.

Seven weeks after gene excision, KO mice exhibit a range of electrophysiologic dysfunction as measured by telemetered ECG. The most pronounced effects are slowed ventricular activation observed as increased QRS width (*p* < 0.01, Figure 1A, left), and a much more prominent and positive-going T-wave than is normally measurable via standard lead II recordings in the mouse (*p* < 0.0001), particularly during epinephrine challenge (Figures 1B,C). Interestingly, these changes exist in the absence of significant rate-corrected QT prolongation (*p* = 0.07, Figure 1A, right), thus suggesting that terminal repolarization is not delayed in the KO mice. These outcomes will be discussed in more detail later, but are consistent with a markedly altered AP morphology in the KO myocytes.

The combined influence of these electrocardiographic abnormalities did not result in a higher frequency of either isolated PVCs or runs of non-sustained (or sustained) VT (*p* > 0.1). In fact, during Epi challenge we observed reduced frequency of PVCs (*p* < 0.05) and a tendency for reduced NSVT frequency in the KO group (*p* = 0.09, Figure 1D). Together, these observations suggest that while the KO mice exhibit clear disturbances to global cardiac electrophysiology, they remain resilient to arrhythmia.

### SERCA2-KO Myocytes Have Prolonged Action Potentials Due to Markedly Increased Sarcolemmal Ca^2+^ Fluxes

**FIGURE 2:**
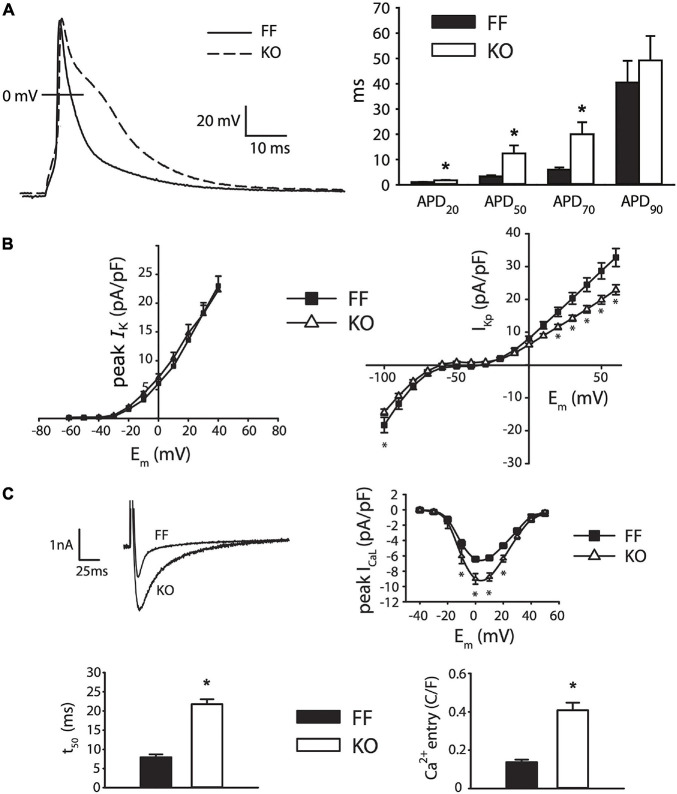
Myocytes from failing SERCA2-KO hearts exhibit prolonged early repolarization due to exaggerated sarcolemmal calcium flux. **(A)** KO myocytes (*n* = 10) display marked prolongation of early repolarization compared to FF (*n* = 11), although this difference is normalized by 90% repolarization. **(B)** The dominant outward current during early repolarization (*I*_to_), is unaltered in the failing KO myocytes relative to FF (*n* = 10, both groups; left panel), while slowly inactivating K^+^ current components (measured as end-pulse, “pedestal” or “plateau,” K^+^ current – *I*_Kp_) were slightly suppressed in KO cells (*n* = 17) relative to FF (*n* = 20). **(C)** However, peak *I*_CaL_ is potentiated and *I*_CaL_ inactivation is dramatically slowed in KO cells (*n* = 14) versus FF (*n* = 12), leading to a marked increase in calcium influx. All panels: *p < 0.05. Data are means ± SEM.

Figure 2 shows the effect of SERCA2 loss on the cardiomyocyte AP and underlying currents. At the level of the AP (Figure 2A), the outstanding feature is a brief early plateau in KO myocytes, which is reminiscent of the phase 2 plateau in large mammals albeit much shorter (∼20 ms). Mechanistically, this plateau indicates that the balance of currents in early repolarization is shifted inwardly, particularly in the region between +20 and −20 mV. This effect was pronounced, and can be seen as a 273% longer APD_50_ and 230% longer APD_70_ in KO myocytes (both *p* < 0.01). Panels B and C show that this effect is due to modest potentiation of peak *I*_CaL_ (39% increase at 0 mV, *p* < 0.001), and dramatically slowed *I*_CaL_ inactivation (50% relaxation time at 0 mV is increased by 178% in KO, *p* < 0.001), which together result in a 200% increase in Ca^2+^ influx, measured as the *I*_CaL_ integral during the square-pulse voltage clamp protocol (*p* < 0.001). Exacerbating this gain in *I*_CaL_ was a moderate reduction of end-pulse potassium current (*I*_Kp_) at positive potentials (*p* < 0.05). While we did not attempt to experimentally dissect the different components of this current, we used a computational model to assess the ability of those components to contribute to the observed changes in AP morphology. These analyses are described in further detail below.

**FIGURE 3:**
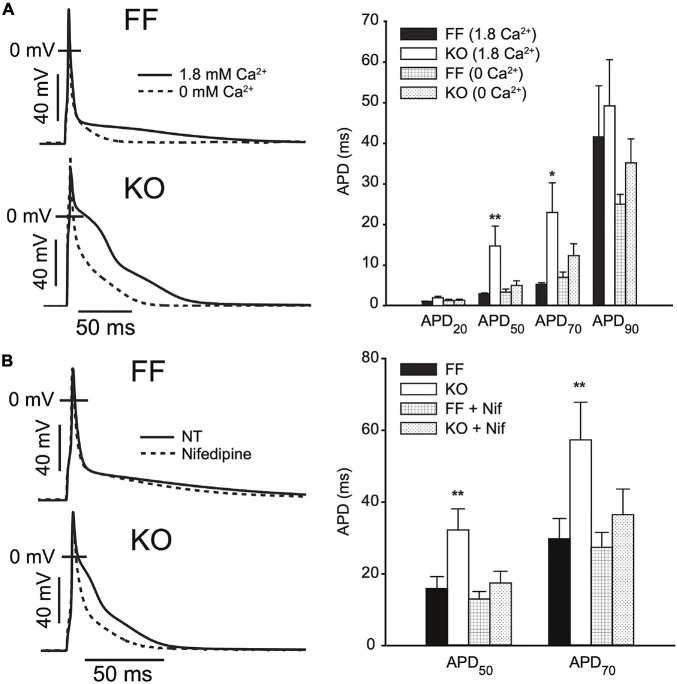
AP prolongation in SERCA2-KO myocytes relies upon augmented L-type calcium current. **(A)** Superfusing FF (*n* = 7) and KO (*n* = 8) myocytes with nominally Ca^2+^ free extracellular solution abolishes early AP prolongation in KO myocytes, while elevating extracellular Ca^2+^ to 1.8 mM exaggerates the difference in APD_50_ and APD_70_ (**p < 0.05 FF vs. KO at 1.8 mM Ca^2+^; and *p* < 0.05 KO at 1.8 mM vs. 0 mM Ca^2+^, *p < 0.0 KO at 1.8 mM vs. 0 mM Ca^2+^). **(B)** *I*_CaL_ blockade via Nifedipine (1 μM) achieves similar normalization of APD_50_ and APD_70_ in KO myocytes (*n* = 7) relative to FF (*n* = 7). **p < 0.05 FF vs. KO in control superfusate; and *p* < 0.05 KO in control vs. Nifedipine. All panels: data are means ± SEM.

In Figure 3 we elaborate on the role of *I*_CaL_ in slowing early repolarization either by acutely changing superfusate Ca^2+^ concentration, or applying *I*_CaL_ blockade by Nifedipine (Nif, 1 μM). Figure 3A shows that the difference in APD_50_ and APD_70_ is removed when Ca^2+^ is excluded from the bath, and slightly exaggerated when it is increased to 1.8 mM (both *p* < 0.05). These alterations in extracellular [Ca^2+^] did not have statistically discernible effects on the kinetics of early repolarization in FF myocytes (*p* > 0.1). Because field-screening effects and non LCC-specific Ca fluxes^2+^ (such as Na^+^/Ca^2+^ exchange) will be altered by modulation of extracellular Ca^2+^, we also used Nifedipine to pharmacologically antagonize *I*_CaL_ (Figure 3C). As for removal of extracellular Ca^2+^, this maneuver eliminated virtually all of the delay in early repolarization present in the KO myocytes – APD_50_ and APD_7__0_ were almost completely normalized (both *p* < 0.01 vs. control superfusion). This suggests that inward *I*_CaL_ is the major contributor to the small phase 2 plateau in KO cells. Finally, to determine whether maneuvers that ordinarily modulate Ca^2+^-dependent *I*_CaL_ inactivation (via SR Ca^2+^ release) are ineffective in KO mice, we also assessed the frequency-dependence of peak *I*_CaL_ and *I*_CaL_ inactivation (Supplementary Figure 1). In moving from 0.1 to 1 Hz FF animals exhibit slowed *I*_CaL_ inactivation due to well-known Ca^2+^-dependent facilitation, and this property is dependent on cytosolic Ca^2+^ (Fauconnier et al., 2003). In KO animals this frequency-dependent modulation of *I*_CaL_ inactivation is completely absent (given the already very slow inactivation in KO myocytes), further indicating the loss of SR-dependent *I*_CaL_ control in KO animals.

Importantly, this shift in balance during early repolarization is not mirrored by slowed terminal repolarization. That is, the differences present at APD_50_ and APD_70_ are lost by 90% (APD_90_) repolarization. The dominant inward currents modulating this late phase of repolarization in the mouse are *I*_NaCa_ and recovering *I*_Na_ (Edwards et al., 2014; Morotti et al., 2014). As described further below these inward currents compete with several components of *I*_K_, particularly the inward rectifier K^+^ current (*I*_K1_) to shape terminal repolarization. While we have not directly assessed the balance of these currents during late repolarization in KO myocytes, the near complete absence of triggered Ca^2+^ release (Andersson et al., 2009) drastically reduces the potential for inward *I*_NaCa_. The slower early repolarization would also be expected to limit *I*_Na_ recovery and the potential for *I*_Na_ reactivation.

### EAD Dynamics in SERCA2-KO Myocytes Share Characteristics of Small and Large Mammals

**FIGURE 4:**
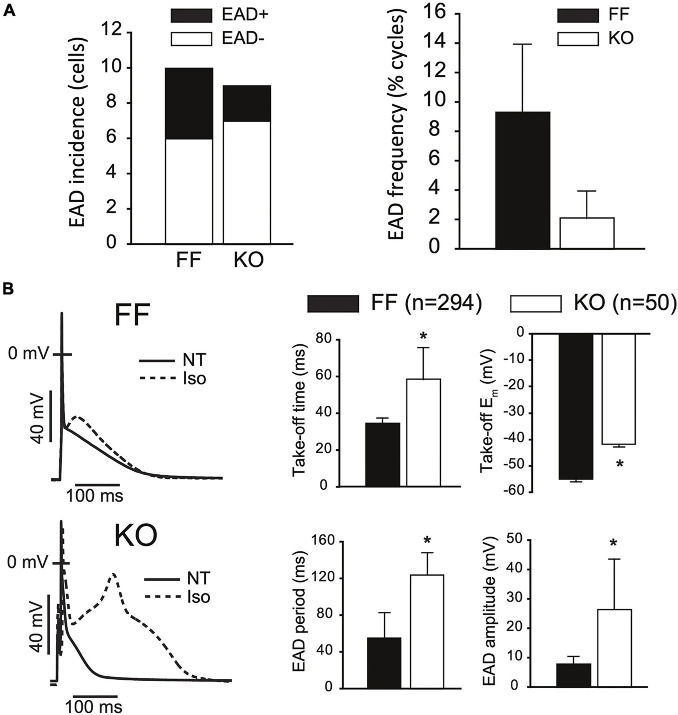
SERCA2-KO myocytes are not more susceptible to EADs but exhibit altered EAD dynamics. **(A)** KO cells (*n* = 9) did not exhibit a higher incidence (left panel: 2/9 KO cells vs. 4/10 FF cells, *p* > 0.01), or frequency (right panel: 50/3037 total KO cycles vs. 293/2593 total FF cycles, *p* > 0.01) of EADs relative to FF (*n* = 10). **(B)** However, KO EADs exhibit altered dynamics as indicated by increased amplitude, period and time to initiation, and reduced (more positive) take-off potentials. All panels: *p < 0.05. Data are means ± SEM.

In larger mammals, the increases in *I*_CaL_ peak current and 50% relaxation time would be expected to promote repolarization instabilities and EADs. To assess EAD susceptibility we challenged KO and FF myocytes with Isoproterenol (1 μM) for at least 3 min while pacing at 1 Hz in current clamp, and defined EADs as any upward deflection in the voltage signal exceeding 3 mV. Figure 4A shows that under these conditions EAD incidence and frequency were not increased in KO cells (both *p* > 0.1), rather EAD frequency tended to be higher (albeit non-significantly, *p* > 0.05) in the FF (9.3 ± 4.7%) than KO group (2.1 ± 1.8%). In FF cells, EADs exhibited signature properties identified in previous work (Sato et al., 2009; Tran et al., 2009). These features included periods with very high APD variability, indicating variable timing of terminal repolarization, but also intermittent beats that did not exhibit EADs. Furthermore, analysis of EAD take-off potentials (Figure 4B) suggests that EADs in the FF group exhibited very similar underlying dynamics to those we have described previously in the mouse, and different to those in larger mammals (Edwards et al., 2014). That is, they initiated at potentials too negative (−55 ± 7 mV) to be carried by *I*_CaL_, and occurred too soon after stimulation (34.6 ± 2.7 ms; approximately coinciding with the peak of the bulk cytosolic Ca^2+^ transient) to result from subcellular Ca^2+^ waves. Thus, it is very likely that EADs in FF myocytes were carried almost entirely by non-equilibrium reactivation of the fast component of the sodium current (*I*_Na_) (Edwards et al., 2014). In contrast, the EADs present in KO myocytes initiated at more positive take-off potentials (−41.8 ± 2 mV, *p* < 0.001), were much larger in amplitude (KO = 26.4 ± 9 mV vs. FF = 7.8 ± 2.6 mV; *p* < 0.001), and lasted much longer than EADs in the FF myocytes (KO = 124 ± 24 ms vs. FF = 55 ± 27 ms; *p* < 0.001). While these EADs initiated later than those in FF cells, because spontaneous SR Ca^2+^ release in KO cells is virtually negligible (Supplementary Figure 2), it is very unlikely that discoordinated SR Ca^2+^ release made any contribution to these events. Even after accounting for leftward shifts in *I*_CaL_ activation due to β-adrenergic activation (Bean et al., 1984; Tiaho et al., 1991; Kumari et al., 2018), the −40 mV take-off potentials are still only just approaching the lower limit of the *I*_CaL_ activation range, thus the ability for *I*_CaL_ to have directly contributed to initiation of these EADs is somewhat limited. However, once initiated, the large amplitude excursions of these EADs (see for example Figure 4B bottom left) suggest that *I*_CaL_ reactivation is pronounced and likely to be the dominant contributor to EAD dynamics, as it is in ventricular myocytes of large mammals (Weiss et al., 2010).

### The Ability for Sarcolemmal Ca^2+^ Fluxes to Shape Murine Repolarization Depends on K^+^ Current Activation Kinetics and Non-equilibrium Na^+^ Current Dynamics

**FIGURE 5:**
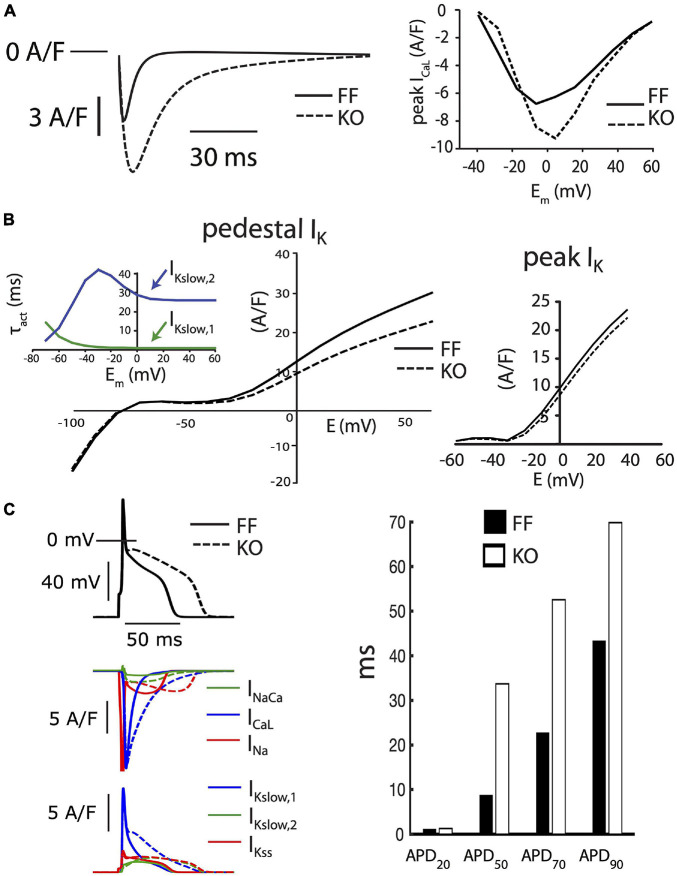
Baseline FF and KO computational models. **(A)** *I*_CaL_ in the KO model was parameterized to permit the markedly slowed *I*_CaL_ inactivation accompanying loss of SR calcium release (left panel) and moderately increased peak current observed in KO myocytes (right panel). **(B)** Conductances for the slower activating component of *I*_Kslow_ (Kv2.1, *I*_Kslow,2_) and the steady state K^+^ current (*I*_Kss_) were both reduced by 25% to fit end-pulse “pedestal” total *I*_K_ (main left panel). Simulated currents were assessed as the sum of all end-pulse K^+^ currents as for the experimental measurements shown in Figure 2, where this compound current is referred to as *I*_Kp_. To simultaneously fit the pedestal and peak components of the compound *I*_K_, as well as differences in *I*_CaL_ and steady state APD, it was necessary to implement the slower activation kinetics of Kv2.1/*I*_Kslow,2_ (inset left). Peak *I*_K_ was straightforwardly fit through scaling the conductance of *I*_to,f_ in both the FF and KO models (right panel). **(C)** Together, these alterations resulted in slowed intermediate repolarization (APD_50_ and APD_70_) similar to that observed for KO myocytes. However, the slower early repolarization and rapid late repolarization in KO cells were much more difficult to capture with models that remained faithful to the voltage-clamp measurements. See Supplementary Table 1 for complete details of differences between these two baseline models.

In considering the balance of currents shaping the trajectory of repolarization in the mouse, an important quantitative aspect is how rapid early repolarization favors *I*_CaL_ deactivation and limits the opportunity for Ca^2+^-dependent *I*_CaL_ inactivation, which is prominent in larger species. Combining this with the known redundancy and different kinetic characteristics among the various K^+^ currents active in this early phase of repolarization, it becomes much more difficult to predict or account for how changes in *I*_CaL_ regulation can contribute to modulating early repolarization. To interrogate these dynamics, we employed a published computational model of the mouse cardiomyocyte, which is specifically parameterized to incorporate the reduced SR Ca^2+^ handling and altered Na^+^ balance in failing KO myocytes [16]. This model already incorporates the increased Cav1.2 and NCX1 expression in KO cells, which leads to exaggerated peak *I*_CaL_ and *I*_NaCa._ However, it does not fully capture the pronounced slowing of *I*_CaL_ inactivation we have observed in KO myocytes at 7 weeks of age, and incorporating this characteristic was our first alteration. Figure 5A shows the behavior of the reparameterized *I*_CaL_ model in square-pulse voltage-clamp protocols identical to our experiments (all parameter changes are provided in full in Supplementary Table 1). Even for its larger peak current and much slower inactivation, this *I*_CaL_ model was still overwhelmed by the fast component of the transient outward current (*I*_to,f_) during early repolarization in both the FF and KO models, and had relatively little impact on the trajectory of early repolarization (APD_20_, Figure 5C).

As mentioned above, KO myocytes also exhibit a reduction in the measurable total *I*_K_ at the end of our 500 ms test steps. The molecular identity of K^+^ channels contributing to this slowly inactivating current are not fully reconciled, although it is clear that the slow component of the transient outward current (carried by the Kv1.4 alpha subunit) is minimally expressed in mice, and that currents carried by Kv1.5 and Kv2.1 make important contributions (Nerbonne, 2014). Together these channels carry the slowly inactivating current (*I*_Kslow_), also commonly referred to as the ultrarapidly activating delayed rectifier (*I*_Kur_) K^+^ current. *I*_Kslow_ is a dominant contributor to the end-pulse K^+^ current in mice. It combines with the steady state K^+^ current (*I*_Kss_, primarily carried by TASK1 and TREK1 channels (Nerbonne, 2014), and *I*_K1_ at more negative potentials, to comprise the compound end-pulse *I*_K_ that we term the “pedestal” or “plateau” *I*_Kp_. Due to difficulties in separating the activation kinetics of the Kv1.5 and Kv2.1 contributions to *I*_Kslow_ in cardiac cells, most models have assumed that this current is a single functional entity, with very rapid activation and slow inactivation. One relatively recent model separated the currents for the purpose of implementing differing phosphoregulation and inactivation kinetics (Morotti et al., 2014). To simultaneously fit intermediate AP prolongation (APD_50_ and APD_70_) and measured end pulse *I*_K_ with the KO model, we also had to employ the approach of Morotti et al. (2014) and separate *I*_Kslow_ into Kv1.5-(*I*_Kslow,1_) and Kv2.1-(*I*_Kslow,2_) specific components. However, unlike Morotti et al. (2014) we implemented slower activation kinetics for *I*_Kslow,2_, as shown in Figure 5B, and which can be measured for Kv2.1 in heterologous systems (Gordon et al., 2006). These slower activation kinetics of Kv2.1 slowed the onset of *I*_Kslow_ enough to permit *I*_CaL_ modulation of intermediate repolarization, while also matching end-pulse *I*_K_ and permitting stable overall repolarization, as observed in our experiments.

### EAD Dynamics in KO Myocytes Are a Mix of Mouse EAD Dynamics and Larger Mammal EAD Dynamics

**FIGURE 6:**
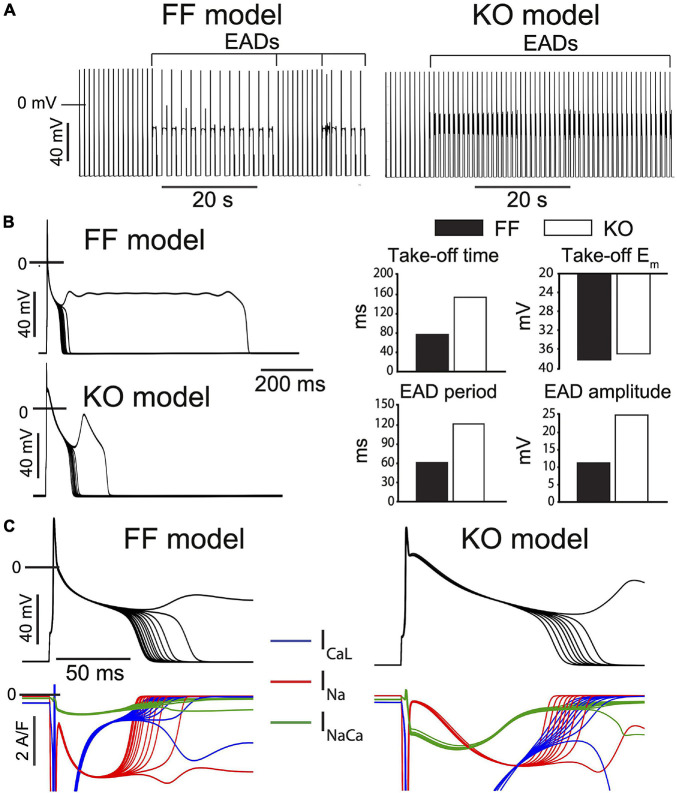
Progressive β-adrenergic challenge was simulated in both the FF and KO models [13] and recapitulate the major differences in EAD dynamics observed experimentally. **(A)** Both models transition to an unstable repolarization regime involving EADs. **(B)** Analysis of the EAD oscillations suggest the model dynamics are similar to those observed experimentally, where EAD amplitude, period and time of initiation are all increased in KO cells, while take-off potential is only slightly more positive. **(C)** Somewhat surprisingly, EAD initiation and the transition into the unstable regime is driven by non-equilibrium reactivation of *I*_Na_ rather than reactivation of *I*_CaL_ in both models, although this dominant role for *I*_Na_ is slightly more clear for the FF model.

We employed mathematical models specific to the KO and FF myocytes (Li et al., 2012) and challenged them with a simple model implementation of β-adrenergic stimulation, which is closely analogous to our experimental challenge. This strategy involves implementing established effects of β-adrenergic regulation at *I*_CaL_ (2–3 fold increased whole-cell permeability, P_CaL_, and 5–10 mV left-shifted steady state activation), SERCA (50–60% reduction in the K_m_ for cytosolic Ca^2+^ binding), the Na^+^-K^+^ ATPase (25–30% reduction in the K_m_ for cytosolic Na^+^ binding), and *I*_Kur_ (15–20% increase in whole cell conductance) (Edwards et al., 2014). The steady-state baseline models for the FF and KO myocytes were each simulated for 3 min at 1 Hz after applying these β-adrenergic parameter changes. Via this approach, EADs could be initiated in both the FF and KO models with identical changes to these key parameters, which remained wholly within the physiologic range. The EADs that result share several of the key features of EADs observed in our experiments during saturating Isoproterenol challenge. First the KO EADs exhibit greater *I*_CaL_ reactivation, and their dynamical characteristics (oscillation period and amplitude) are increased similarly to the experimental recordings (Figure 4B). These dynamic characteristics are similar to those of *I*_CaL_-dominated EADs in large mammals (Sato et al., 2010). However, importantly, and somewhat surprisingly, even though the model EADs occurred at relatively positive potentials (approximately −38 mV) and relatively late (80 to 120 ms after the AP stimulus), they were initiated in both models by non-equilibrium reactivation of *I*_Na_ (Edwards et al., 2014) rather than *I*_CaL_ (Figure 6C). This suggests that even for EADs that initiate 120 ms after the AP peak, recovery from inactivation of *I*_Na_ is sufficient to permit an arrhythmogenic current reactivation.

## Discussion

The ability for SR Ca^2+^ release to act as a cellular driver of cardiac arrhythmia has been appreciated for at least 30 years (Lederer and Tsien, 1976), and is widely recognized as a mechanistic contributor to all major cellular arrhythmogenic behaviors (automaticity, EADs, DADs, and APD alternans). The relative importance of SR Ca^2+^ fluxes in each behavior is highly dependent on the electrophysiologic context, and in this study we highlight species-specificity. By studying the electrophysiologic characteristics of murine SERCA2 knock out, we have assessed the ability for membrane versus SR mechanisms to generate arrhythmia upon the background of the characteristically large repolarizing currents in the mouse. We report four findings that are likely to hold important implications for other studies assessing electrophysiologic outcomes in mice, both in health and disease: (1) Shifting the burden of contractile calcium flux away from the SR and toward the sarcolemma elicits a pronounced, positive, and prolonged T-wave that is not normally observable in mice. While these effects are substantial, they are not sufficient to destabilize cardiac activation or repolarization. (2) The major cellular effect underlying these global changes is a very large increase in *I*_CaL_-mediated Ca^2+^ influx, due largely to slowed current inactivation, and which results in a pronounced delay of early repolarization creating an unusual AP plateau around 0 mV. (3) This slowing of early repolarization is difficult to capture in models due to the rapid kinetics of the large measurable peak *I*_K_, which is broadly thought to be carried by *I*_to,f_ and *I*_Kur_/*I*_Kslow,1_. The manner in which K^+^ current activation interacts with *I*_CaL_ inactivation to shape early repolarization of the murine AP in this way requires further review. (4) The overall shift in the balance of membrane currents in SERCA2-KO away from a mouse-like phenotype and toward a large mammalian phenotype recruits EAD dynamics that are closer to those in large mammals. However, this shift is not sufficient to increase EAD frequency or incidence, and initiation of infrequent EADs in KO myocytes still likely relies upon *I*_Na_ reactivation, which rarely drives EADs in large mammalian myocytes.

The appearance of gross ECG changes in KO mice, particularly in T-wave amplitude, was remarkable and somewhat surprising given that the T-wave is often virtually absent in mice (Liu et al., 2004). However, these effects are captured by other models of pronounced induction of *I*_CaL_ in mice and rats. Early rat studies of agonist dihydropiridines (Bay K 8644), which both potentiate and markedly prolong *I*_CaL,_ noted similar overall changes and included additional ST segment elevation due to fusion of the QRS complex and T-wave (Abraham et al., 1987). Similarly in the mouse, β-adrenergic stimulation elicits a roughly twofold increase in T-wave amplitude (0.16 mV), and significant dispersion of repolarization as measured by T-wave decay time (Speerschneider and Thomsen, 2013). Finally here, one genetic model with very similar changes to macroscopic *I*_CaL_ is Timothy syndrome (formerly type 8 long QT syndrome). While mice have been generated for major mutations known to cause this channelopathy, and brief reports suggest QT prolongation and abnormal ECG characteristics (Bett et al., 2009), we are unaware of any comprehensive description of the changes in cardiac electrophysiology present in this mouse.

Our prior work with the SERCA2-KO mouse has clearly shown that 7 weeks after genetic ablation little to no SERCA expression remains, and the SR Ca^2+^ store is essentially eliminated (Andersson et al., 2009; Louch et al., 2010b). To verify that spontaneous SR Ca^2+^ release is similarly absent in the 7-week KO mice, we also measured Ca^2+^ sparks and waves in isolated myocytes and observed that both were essentially abolished (Supplementary Figure 2). This is key in the context of the arrhythmogenic outcomes of interest here because it eliminates at least one major class of triggered arrhythmia resulting from spontaneous Ca^2+^ release and DADs. With respect to EADs, the implications of SERCA-KO are more complex. Previous studies have suggested that SR Ca^2+^ release is involved in murine EADs (Pott et al., 2012; Edwards et al., 2014). Specifically, we have shown that exaggerated SR Ca^2+^ release is the critical proximal mechanism of slowed late repolarization in mouse ventricle, and that this in turn elicits EADs through non-equilibrium reactivation of *I*_Na_ (Edwards et al., 2014). By this mechanism it is predictable that EADs and their arrhythmogenic potential would be inhibited in the KO mice. However, the established and very large increases in sarcolemmal Ca^2+^ transport in these mice create a very different electrophysiologic context. One which shifts repolarization dynamics toward those present in larger mammalian myocytes, and in principle may predispose to EADs with large mammal-like dynamics. We have shown here that this remarkable degree of plasticity is not sufficient to induce such instability.

Two important examples of genetic mouse models that exhibit very different adaptations to the SERCA-KO mouse are provided by murine knock-out and transgenic overexpression of the cardiac Na^+^-Ca^2+^ exchanger (NCX1). Henderson et al. (2004) developed a cardiac-specific NCX1 knock-out (NCX1-KO) mouse that lives to adulthood and exhibits 80-90% reduction in NCX1 expression. Through a set of (virtually opposite) adaptations to those resulting from SERCA-KO, the NCX1-KO mouse also retains remarkably viable contractile function. Unlike the SERCA-KO mice, these animals exhibit much reduced sarcolemmal Ca^2+^ transport but achieve sufficient contractile activation through very high gain EC coupling (Pott et al., 2005). While they exhibit clearly shortened APs (largely due to increased *I*_to,f_) and QT intervals, they do not present an overt arrhythmia phenotype (Pott et al., 2007). In contrast, the NCX1-transgenic mouse developed by the same group exhibits pronounced slowing of terminal repolarization (prolonged APD_90_), EADs, and an overt susceptibility to arrhythmia (Pott et al., 2012). Taken together, these studies strongly support the remarkable ability of cardiomyocytes to autonomously reconfigure their Ca^2+^ handling and electrophysiologic machinery to support the Ca^2+^ requirements for contraction. However, they also suggest that the gene regulatory programs governing those reconfigurations are not equally apt to defend electrophysiologic stability. That is, in response to certain perturbations, the regulatory adjustments made to defend contractile Ca^2+^ will be arrhythmogenic. Our SERCA-KO mouse can be considered fortunate in this respect.

## Limitations

One immediately observable characteristic of the KO myocyte AP is marked slowing of early repolarization between +20 and −20 mV. While we did not attempt to broadly reparametrize the kinetics of the *I*_to,f_ model to fit this characteristic, it proved challenging to reconcile with our peak *I*_K_ measurements, which readily overwhelmed even the large changes in peak *I*_CaL_ and *I*_CaL_ inactivation. While we are unaware of other studies that have systematically investigated the ability for altered *I*_CaL_ inactivation kinetics to prolong this early portion of the murine AP, this characteristic has been clearly observed in previous studies. For example, acutely evacuating the SR store via caffeine both markedly prolongs early repolarization and accelerates later repolarization in a manner that is remarkably consistent with the AP morphology in KO myocytes [see Figure 3C in Edwards et al., 2014)]. Because the dynamics of interaction between *I*_CaL_ and *I*_K_ during this phase of the AP are critical for determining Ca^2+^ influx, we suggest that they deserve further investigation.

Additionally, we had limited ability to reconcile the more rapid terminal repolarization (APD_70_ to APD_90_) in KO myocytes while constraining *I*_K1_ to the measured inward end-pulse current below *E*_K_ (Figure 2B, right panel). We (Edwards et al., 2014) and others (Yao et al., 1998; Pott et al., 2007, 2012; Ferreiro et al., 2012) have shown that SR Ca^2+^ release and resulting inward *I*_NaCa_ are major modulators of this phase of the murine AP. This suggests that more detailed accounting for components of *I*_K_ that are active and available between −80 and −40 mV (particularly *I*_K1_), is likely necessary for properly capturing the interaction among *I*_NaCa_, recovering *I*_Na_ and *I*_K_ in determining the trajectory of terminal repolarization in the mouse.

## Conclusion

The major shifts in sarcolemmal Ca^2+^ transport resulting from cardiac specific SERCA2 knock out are clearly not sufficient to destabilize myocyte repolarization or global electrophysiology in the mouse. Furthermore, those shifts only alter EAD dynamics in a relatively subtle fashion. In sum, our data suggest that, in the mouse, the destabilizing influence of extreme changes to sarcolemmal Ca^2+^ influx and extrusion can be readily outweighed by the stabilizing influence of an absent SR Ca^2+^ store and dramatic reduction in spontaneous SR Ca^2+^ release. Of course, this characteristic relies on the very strong overall repolarization present in the mouse, and should not be interpreted to extend to humans or indeed any larger mammals exhibiting an AP plateau.

## Data Availability Statement

The raw data supporting the conclusions of this article will be made available by the authors, without undue reservation.

## Ethics Statement

The animal study was reviewed and approved by the Norwegian National Committee for Animal Welfare.

## Author Contributions

AGE collected and analyzed data, performed computational analyses, conceived and designed aspects of the study, and wrote the manuscript. HM collected and analyzed data, conceived and designed aspects of the study, and reviewed the manuscript. MKS collected and analyzed data, and reviewed the manuscript. DBL collected and analyzed data. IS conceived and designed aspects of the study, and reviewed the manuscript. SR and WEL collected and analyzed data, conceived and designed aspects of the study, reviewed the manuscript, and contributed to funding the study. OMS conceived and designed aspects of the study, reviewed the manuscript, and contributed to funding the study. All authors contributed to the article and approved the submitted version.

## Conflict of Interest

The authors declare that the research was conducted in the absence of any commercial or financial relationships that could be construed as a potential conflict of interest.

## Publisher’s Note

All claims expressed in this article are solely those of the authors and do not necessarily represent those of their affiliated organizations, or those of the publisher, the editors and the reviewers. Any product that may be evaluated in this article, or claim that may be made by its manufacturer, is not guaranteed or endorsed by the publisher.

## References

[B1] Abraham S. Amitai G. Oz N. Weissman B. A. (1987). Bay K 8644-induced changes in the ECG pattern of the rat and their inhibition by antianginal drugs. *Br. J. Pharmacol.* 92 603–608. 10.1111/j.1476-5381.1987.tb11362.x 2447988 PMC1853702

[B2] Andersson K. B. Birkeland J. A. Finsen A. V. Louch W. E. Sjaastad I. Wang Y. (2009). Moderate heart dysfunction in mice with inducible cardiomyocyte-specific excision of the Serca2 gene. *J. Mol. Cell. Cardiol.* 47:187. 10.1016/j.yjmcc.2009.03.013 19328205

[B3] Bai Y. Jones P. P. Guo J. Zhong X. Clark R. B. Zhou Q. (2013). Phospholamban knockout breaks arrhythmogenic Ca^2 +^ ; waves and suppresses catecholaminergic polymorphic ventricular tachycardia in mice. *Circ. Res.* 113 517–526. 10.1161/circresaha.113.301678 23856523 PMC3864692

[B4] Bean B. P. Nowycky M. C. Tsien R. W. (1984). β-Adrenergic modulation of calcium channels in frog ventricular heart cells. *Nature* 307 371–375. 10.1038/307371a0 6320002

[B5] Bers D. M. (2001). Excitation-contraction coupling and cardiac contractile force. *J. Cardiova. Dis. Res.* 1:45.

[B6] Bett G. C. L. Bondarenko V. E. Rasmusson R. L. (2009). Cardiac characteristics of a mouse model of timothy syndrome. *Biophys. J.* 96 665a–666a.

[B7] Brunner M. Peng X. Liu G. X. Ren X. Q. Ziv O. Choi B. R. (2008). Mechanisms of cardiac arrhythmias and sudden death in transgenic rabbits with long QT syndrome. *J. Clin. Invest.* 118 2246–2259. 18464931 10.1172/JCI33578 PMC2373420

[B8] Cerrone M. Noujaim S. F. Tolkacheva E. G. Talkachou A. O’Connell R. Berenfeld O. (2007). Arrhythmogenic mechanisms in a mouse model of catecholaminergic polymorphic ventricular tachycardia. *Circ. Res.* 101 1039–1048. 17872467 10.1161/CIRCRESAHA.107.148064 PMC2515360

[B9] Edwards A. G. Grandi E. Hake J. E. Patel S. Li P. Miyamoto S. (2014). Nonequilibrium reactivation of Na+ current drives early afterdepolarizations in mouse ventricle. *Circ. Arrhyt. Electrophys.* 7:1213. 10.1161/CIRCEP.113.001666 PMC4301603 25236710

[B10] Fauconnier J. Bedut S. Guennec J.-Y. L. Babuty D. Richard S. (2003). Ca^2 +^ current-mediated regulation of action potential by pacing rate in rat ventricular myocytes. *Cardiovasc. Res.* 57 670–680. 10.1016/s0008-6363(02)00731-9 12618229

[B11] Fearnley C. J. Roderick H. L. Bootman M. D. (2011). Calcium signaling in cardiac myocytes. *CSH Perspect. Biol.* 3:a004242. 10.1101/cshperspect.a004242 PMC3220352 21875987

[B12] Ferreiro M. Petrosky A. D. Escobar A. L. (2012). Intracellular Ca^2 +^ release underlies the development of phase 2 in mouse ventricular action potentials. *Am. J. Physiol. Heart C* 302 H1160–H1172. 10.1152/ajpheart.00524.2011 PMC3311451 22198177

[B13] Gordon E. Roepke T. K. Abbott G. W. (2006). Endogenous KCNE subunits govern Kv2.1K+ channel activation kinetics in xenopus oocyte studies. *Biophys. J.* 90 1223–1231. 10.1529/biophysj.105.072504 16326911 PMC1367273

[B14] Henderson S. A. Goldhaber J. I. So J. M. Han T. Motter C. Ngo A. (2004). Functional adult myocardium in the absence of Na+-Ca^2 +^ exchange. *Circ. Res.* 95 604–611. 15308581 10.1161/01.RES.0000142316.08250.68

[B15] Kashimura T. Briston S. J. Trafford A. W. Napolitano C. Priori S. G. Eisner D. A. (2010). In the RyR2R4496C mouse model of CPVT, β-adrenergic stimulation induces CA waves by increasing SR Ca content and not by decreasing the threshold for Ca waves. *Circ. Res.* 107 1483–1489. 10.1161/circresaha.110.227744 20966392

[B16] Koren G. (2009). Electrical remodeling and arrhythmias in long-QT syndrome: lessons from genetic models in mice. *Ann. Med.* 36 22–27. 10.1080/17431380410032643 15176420

[B17] Kumari N. Gaur H. Bhargava A. (2018). Cardiac voltage gated calcium channels and their regulation by β-adrenergic signaling. *Life Sci.* 194 139–149. 10.1016/j.lfs.2017.12.033 29288765

[B18] Land S. Louch W. E. Niederer S. A. Aronsen J. M. Christensen G. Sjaastad I. (2013). Beta-adrenergic stimulation maintains cardiac function in serca2 knockout mice. *Biophys. J.* 104 1349–1356. 10.1016/j.bpj.2013.01.042 23528094 PMC3602781

[B19] Lederer W. J. Tsien R. W. (1976). Transient inward current underlying arrhythmogenic effects of cardiotonic steroids in Purkinje fibres. *J. Physiol.* 263 73–100. 10.1113/jphysiol.1976.sp011622 1018270 PMC1307691

[B20] Lehnart S. E. Mongillo M. Bellinger A. Lindegger N. Chen B. X. Hsueh W. (2008). Leaky Ca^2 +^ release channel/ryanodine receptor 2 causes seizures and sudden cardiac death in mice. *J. Clin. Invest.* 118 2230–2245. 18483626 10.1172/JCI35346 PMC2381750

[B21] Lehnart S. E. Terrenoire C. Reiken S. Wehrens X. H. Song L. S. Tillman E. J. (2006). Stabilization of cardiac ryanodine receptor prevents intracellular calcium leak and arrhythmias. *Proc. Natl. Acad. Sci. U.S.A.* 103:7910. 10.1073/pnas.0602133103 PMC1472543 16672364

[B22] Li L. Louch W. E. Niederer S. A. Aronsen J. M. Christensen G. Sejersted O. M. (2012). Sodium accumulation in SERCA knockout-induced heart failure. *Biophys. J.* 102:2048. 10.1016/j.bpj.2012.03.045 PMC3341551 22824267

[B23] Liu G. Iden J. B. Kovithavongs K. Gulamhusein R. Duff H. J. Kavanagh K. M. (2004). In vivo temporal and spatial distribution of depolarization and repolarization and the illusive murine T wave. *J. Physiol.* 555 267–279. 10.1113/jphysiol.2003.054064 14634200 PMC1664824

[B24] Liu X. H. Zhang Z. Y. Andersson K. B. Husberg C. Enger U. H. Ræder M. G. (2011). Cardiomyocyte-specific disruption of Serca2 in adult mice causes sarco(endo)plasmic reticulum stress and apoptosis. *Cell Calcium* 49 201–207. 10.1016/j.ceca.2010.09.009 20965565

[B25] Louch W. E. Hake J. Jølle G. F. Mørk H. K. Sjaastad I. Lines G. T. (2010a). Control of Ca^2 +^ release by action potential configuration in normal and failing murine cardiomyocytes. *Biophys. J.* 99 1377–1386. 10.1016/j.bpj.2010.06.055 20816049 PMC2931738

[B26] Louch W. E. Hougen K. Mørk H. K. Swift F. Aronsen J. M. Sjaastad I. (2010b). Sodium accumulation promotes diastolic dysfunction in end-stage heart failure following Serca2 knockout. *J. Physiol.* 588:478. 10.1113/jphysiol.2009.183517 PMC2825611 20008467

[B27] Manotheepan R. Danielsen T. K. Sadredini M. Anderson M. E. Carlson C. R. Lehnart S. E. (2016). Exercise training prevents ventricular tachycardia in CPVT1 due to reduced CaMKII-dependent arrhythmogenic Ca 2+ release. *Cardiovasc. Res.* 111 295–306. 10.1093/cvr/cvw095 27161030 PMC4957490

[B28] Mørk H. K. Sjaastad I. Sejersted O. M. Louch W. E. (2009). Slowing of cardiomyocyte Ca^2 +^ release and contraction during heart failure progression in postinfarction mice. *Am. J. Physiol. Heart C* 296 H1069–H1079. 10.1152/ajpheart.01009.2008 19201998

[B29] Morotti S. Edwards A. G. McCulloch A. D. Bers D. M. Grandi E. (2014). A novel computational model of mouse myocyte electrophysiology to assess the synergy between Na+ loading and CaMKII. *J. Physiol.* 592 1181–1197. 10.1113/jphysiol.2013.266676 24421356 PMC3961080

[B30] Nerbonne J. M. (2014). Mouse models of arrhythmogenic cardiovascular disease: challenges and opportunities. *Curr. Opin. Pharmacol.* 15 107–114. 10.1016/j.coph.2014.02.003 24632325 PMC3984610

[B31] Ottesen A. H. Louch W. E. Carlson C. R. Landsverk O. J. B. Kurola J. Johansen R. F. (2015). Secretoneurin is a novel prognostic cardiovascular biomarker associated with cardiomyocyte calcium handling. *J. Am. Coll. Cardiol.* 65:351. 10.1016/j.jacc.2014.10.065 25634832

[B32] Piot C. LeMaire S. A. Albat B. Seguin J. Nargeot J. Richard S. (1996). High frequency–induced upregulation of human cardiac calcium currents. *Circulation* 93 120–128. 10.1161/01.cir.93.1.120 8616918

[B33] Pogwizd S. M. Bers D. M. (2002). Calcium cycling in heart failure: the arrhythmia connection. *J. Cardiovasc. Electr.* 13 88–91. 10.1046/j.1540-8167.2002.00088.x 11843491

[B34] Pogwizd S. M. Schlotthauer K. Li L. Yuan W. Bers D. M. (2001). Arrhythmogenesis and contractile dysfunction in heart failure. *Circ. Res.* 88 1159–1167. 10.1161/hh1101.091193 11397782

[B35] Pott C. Muszynski A. Ruhe M. Bögeholz N. Schulte J. S. Milberg P. (2012). Proarrhythmia in a non-failing murine model of cardiac-specific Na+/Ca 2+ exchanger overexpression: whole heart and cellular mechanisms. *Basic. Res. Cardiol.* 107:247. 10.1007/s00395-012-0247-7 PMC3500087 22327339

[B36] Pott C. Philipson K. D. Goldhaber J. I. (2005). Excitation–contraction coupling in Na+–Ca^2 +^ exchanger knockout mice. *Circ. Res.* 97 1288–1295. 16293789 10.1161/01.RES.0000196563.84231.21 PMC1790864

[B37] Pott C. Ren X. Tran D. X. Yang M. J. Henderson S. Jordan M. C. (2007). Mechanism of shortened action potential duration in Na+-Ca^2 +^ exchanger knockout mice. *Am. J. Physiol. Cell.* 292 C968–C973. 10.1161/01.res.0000196563.84231.21 16943244

[B38] Priori S. G. Napolitano C. Memmi M. Colombi B. Drago F. Gasparini M. (2002). Clinical and molecular characterization of patients with catecholaminergic polymorphic ventricular tachycardia. *Circulation* 106 69–74. 12093772 10.1161/01.cir.0000020013.73106.d8

[B39] Priori S. G. Napolitano C. Tiso N. Memmi M. Vignati G. Bloise R. (2001). Mutations in the cardiac ryanodine receptor gene (hRyR2) underlie catecholaminergic polymorphic ventricular tachycardia. *Circulation* 103 196–200. 10.1161/01.cir.103.2.196 11208676

[B40] Sato D. Xie L. H. Sovari A. A. Tran D. X. Morita N. Xie F. (2009). Synchronization of chaotic early afterdepolarizations in the genesis of cardiac arrhythmias. *Proc. Natl. Acad. Sci. U.S.A.* 106:2988. 10.1073/pnas.0809148106 PMC2651322 19218447

[B41] Sato D. Xie L.-H. Nguyen T. P. Weiss J. N. Qu Z. (2010). Irregularly appearing early afterdepolarizations in cardiac myocytes: random fluctuations or dynamical chaos? *Biophys. J.* 99:773. 10.1016/j.bpj.2010.05.019 PMC2913181 20682253

[B42] Shannon T. R. Wang F. Puglisi J. Weber C. Bers D. M. A. (2004). Mathematical treatment of integrated ca dynamics within the ventricular myocyte. *Biophys J.* 87 3351–3371. 10.1529/biophysj.104.047449 15347581 PMC1304803

[B43] Speerschneider T. Thomsen M. B. (2013). Physiology and analysis of the electrocardiographic T wave in mice. *Acta Physiol.* 209 262–271. 10.1111/apha.12172 24119104

[B44] Splawski I. Timothy K. W. Decher N. Kumar P. Sachse F. B. Beggs A. H. (2005). Severe arrhythmia disorder caused by cardiac L-type calcium channel mutations. *Proc. Natl. Acad. Sci. U.S.A.* 102:8089 8096; discussion 8086-8088. 10.1073/pnas.0502506102 PMC1149428 15863612

[B45] Splawski I. Timothy K. W. Sharpe L. M. Decher N. Kumar P. Bloise R. (2004). Ca(V)1.2 calcium channel dysfunction causes a multisystem disorder including arrhythmia and autism. *Cell* 119:31. 10.1016/j.cell.2004.09.011 15454078

[B46] Swift F. Franzini-Armstrong C. Øyehaug L. Enger U. H. Andersson K. B. Christensen G. (2012). Extreme sarcoplasmic reticulum volume loss and compensatory T-tubule remodeling after Serca2 knockout. *Proc. Natl. Acad. Sci. U.S.A.* 109:4001. 10.1073/pnas.1120172109 PMC3309775 22355118

[B47] Tiaho F. Nargeot J. Richard S. (1991). Voltage-dependent regulation of L-type cardiac Ca channels by isoproterenol. *Pflügers Arch.* 419 596–602. 10.1007/bf00370301 1664936

[B48] Tran D. X. Sato D. Yochelis A. Weiss J. N. Garfinkel A. Qu Z. (2009). Bifurcation and chaos in a model of cardiac early afterdepolarizations. *Phys. Rev. Lett.* 102:258103. 10.1103/PhysRevLett.102.258103 PMC2726623 19659123

[B49] Weiss J. N. Garfinkel A. Karagueuzian H. S. Chen P.-S. Qu Z. (2010). Early afterdepolarizations and cardiac arrhythmias. *Heart Rhythm.* 7 1891–1899. 10.1016/j.hrthm.2010.09.017 20868774 PMC3005298

[B50] Yao A. Su Z. Nonaka A. Zubair I. Lu L. Philipson K. D. (1998). Effects of overexpression of the Na+-Ca^2 +^ Exchanger on [Ca^2 +^ ]i transients in murine ventricular myocytes. *Circ. Res.* 82 657–665. 10.1161/01.res.82.6.657 9546374

