# Supplementary material for: Sarcoplasmic Reticulum Calcium Release Is Required for Arrhythmogenesis in the Mouse
Source: Front Physiol. 2021 Oct 12;12:744730. doi: 10.3389/fphys.2021.744730 (PMC8546347; doi:10.3389/fphys.2021.744730)
Supplement: Supplementary file 1 [file Data_Sheet_1.pdf]

Supplementary materials: Sarcoplasmic reticulum calcium release is required for arrhythmogenesis in the mouse

Supplemental Figure 1.

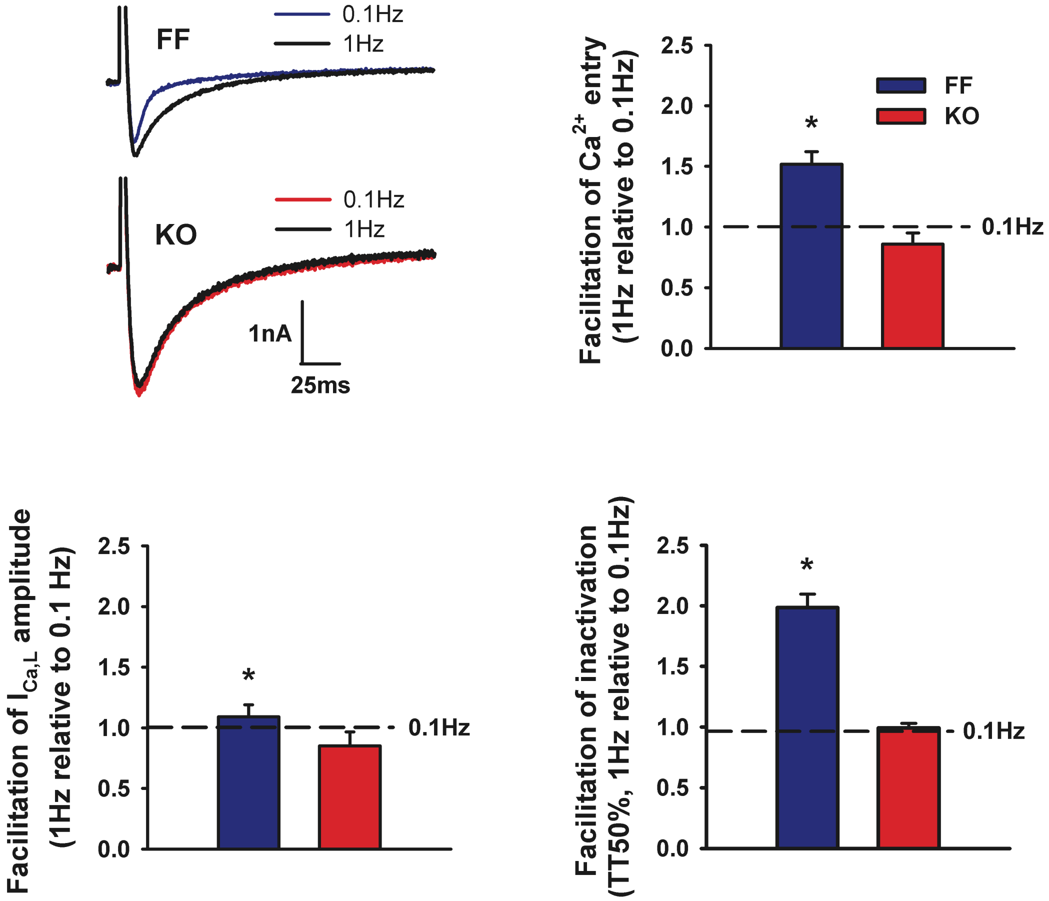

Frequency-dependent I_{CaL} control is eliminated in SERCA2-KO myocytes. While FF cardiomyocytes exhibit robust frequency-dependent I_{CaL} facilitation,[4] the very slow I_{CaL} inactivation present in KO cardiomyocytes remains independent of frequency in the 0.1 – 1 Hz range. Thus, suggesting that voltage-dependent inactivation is the very much the dominant form of I_{CaL} inactivation in these cells. * $p<0.05$ KO vs. FF. Data are shown as means ± SEM.

Supplemental Figure 2.

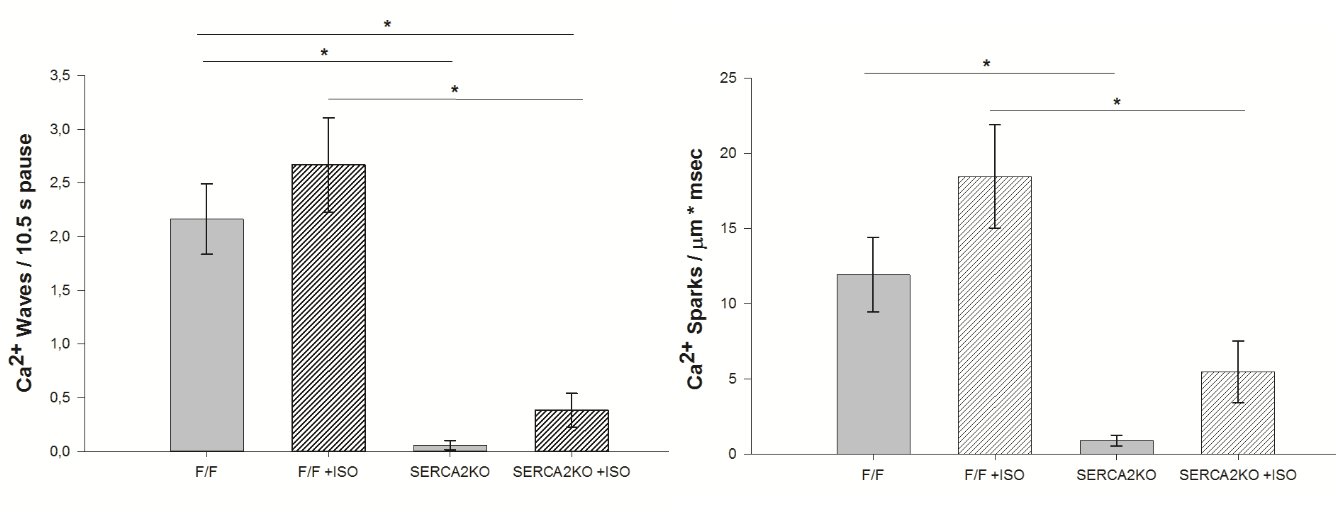

Spontaneous SR calcium release is virtually eliminated in SERCA2-KO myocytes. Under both control conditions and during exposure to 1 μM Isoproterenol, KO myocytes exhibit very few discernible spontaneous Ca^{2+} release events. Ca^{2+} waves were measured via our standard pause-induced release protocols,[2] and similarly for Ca^{2+} sparks.[2,3] All panels: * $p<0.05$ KO vs. FF. Data are shown as means ± SEM.

Computational Modeling:

The general strategy for fitting our I_{CaL}, peak I_K, and end-pulse (pedestal) I_K data is described in the main text. In Supplemental Table 1 we provide all resulting parameter changes that define both the FF and KO Baseline models. We also provide the changes to those parameters that are used to implement acute β-adrenergic challenge. For these β-adrenergic simulations, the FF and KO models were initialized with their steady state conditions resulting from 500 beats at 1 Hz applying the Baseline parameters. As previously, [5] the β-stim parameter changes were then applied at the beginning of a 60-second simulation, during which the resulting impact on the AP and Ca^{2+} handling evolved in time. Models were implemented in Matlab Release 2014b (Mathworks, Natick, MA). All code is publicly accessible.

Supplemental Table 1.

		FF model			KO model		
**Parameter**	**Description**	**Original**	**Baseline**	**β stim**	**Original**	**Baseline**	**β stim**
**C1**	LCC: V-dependent inactivation transition 1 (ms)	33	33	33	33	**38**	38
**C3**	LCC: V-dependent inactivation transition 3 (ms)	0.1	0.1	0.1	0.1	**0.4**	0.4
**C6**	LCC: V-dependent inactivation transition 6 (ms)	5	5	5	10	**40**	40
ϕ_L	LCC: fraction of time closed in open mode (ratio)	2.5	2.5	2.5	2.5	**1.5**	1.5
t_L	LCC: transition latency C to O (ms)	9	9	9	11	**9**	9
V_L	LCC: half maximal LCC activation (mV)	-5	-5	**-14**	-5	-5	**-14**
P_{CaL}	LCC: permeability (μL/ms)	1.90E-07	**1.60E-07**	**4.80E-07**	2.00E-07	**6.00E-08**	**1.80E-07**
$K_{m,up}$	SERCA : Ca^{2+} affinity (μmol/L)	0.4928	0.4928	**0.1971**	0.4928	0.4928	**0.1971**
$g_{Kto,f}$	$I_{to,f}$ maximum conductance (mS/μF)	0.4	**0.35**	0.35	0.4	**0.3**	0.3
g_{Kss}	I_{Kss} maximum conductance (mS/μF)	0.0596	**0.13**	0.13	0.0596	**0.0975**	0.0975
$K_{m,\alpha 1}$	Intracellular Na^+-affinity of NKAα1 isoform (μmol/L)	21000	21000	**15120**	21000	21000	**15120**
$K_{m\alpha 2}$	Intracellular Na^+-affinity of NKAα2 isoform (μmol/L)	21000	21000	**15120**	21000	21000	**15120**
g_{K1}	I_{K1} Maximum conductance (mS/μF)	0.35	**0.9**	0.9	0.35	**0.9**	0.9
g_{Kur}	I_{Kur} Maximum conductance (mS/μF)	0.45	NP	NP	0.45	NP	NP
$g_{Kslow,1}$	$I_{Kslow,1}$ Maximum conductance (mS/μF)	NP	**0.15**	**0.1725**	NP	**0.15**	**0.1725**
$g_{Kslow,2}$	$I_{Kslow,2}$ Maximum conductance (mS/μF)	NP	**0.14**	0.14	NP	**0.105**	0.105

Parameters for models of FF and KO electrophysiology and EC coupling. Parameter values are given for the original FF and KO models as published in Li et al.[6] (**Original**), as well as those for both the FF and KO control models (**Baseline**), and those applied to simulate saturating β-adrenergic stimulation and elicit EADs (**β stim**). Baseline parameter values are **bolded** where they have been altered from the corresponding (FF or KO) Original model. Similarly, bolded β stim parameter values

have been altered from the corresponding Baseline model. Parameter values designated NP indicate those parameters that have been eliminated from the model during revision. This reflects the separation of the original I_{Kur} (I_{Kslow}) into its components ($I_{Kslow,1}$ and $I_{Kslow,2}$).

Supplementary References:

1. Boukens, B. J., Rivaud, M. R., Rentschler, S. & Coronel, R. Misinterpretation of the mouse ECG: 'musing the waves of Mus musculus.' *J Physiology* **592**, 4613–4626 (2014).

2. Manotheepan, R. *et al.* Exercise training prevents ventricular tachycardia in CPVT1 due to reduced CaMKII-dependent arrhythmogenic Ca 2+ release. *Cardiovasc Res* **111**, 295–306 (2016).

3. Kolstad, T. R. *et al.* Ryanodine receptor dispersion disrupts Ca2+ release in failing cardiac myocytes. *Elife* **7**, (2018).

4. Fauconnier, J., Bedut, S., Guennec, J.-Y. L., Babuty, D. & Richard, S. Ca2+ current-mediated regulation of action potential by pacing rate in rat ventricular myocytes. *Cardiovasc Res* **57**, 670–680 (2003).

5. Edwards, A. G. *et al.* Nonequilibrium reactivation of Na+ current drives early afterdepolarizations in mouse ventricle. *Circulation Arrhythmia Electrophysiol* **7**, 1205 1213 (2014).

6. Li, L. *et al.* Sodium accumulation in SERCA knockout-induced heart failure. *Biophys J* **102**, 2039 2048 (2012).
